# A 3D radially aligned nanofiber scaffold co-loaded with LL37 mimetic peptide and PDGF-BB for the management of infected chronic wounds

**DOI:** 10.1016/j.mtbio.2024.101237

**Published:** 2024-09-12

**Authors:** Fei Li, Chuwei Zhang, Xiaoping Zhong, Bo Li, Mengnan Zhang, Wanqian Li, Lifei Zheng, Xinghua Zhu, Shixuan Chen, Yi Zhang

**Affiliations:** a Department of Burn and Plastic Surgery, Affiliated Hospital of Nantong University, Nantong, 226001, China; b Zhejiang Engineering Research Center for Tissue Repair Materials, Wenzhou Institute, University of the Chinese Academy of Sciences, Wenzhou, Zhejiang, 325000, China; c Department of Nursing, Third Affiliated Hospital of Guangzhou Medical University, Guangzhou, Guangdong, 510000, China

**Keywords:** Infected chronic wounds, Antibacterial peptide, Cell recruitment, PDGF-BB, Granulation tissue formation

## Abstract

Diabetic foot ulcers, pressure ulcers, and bedsores can easily develop into chronic wounds with bacterial infections, complicating wound healing. This work reports a two-step strategy for treating infected chronic wounds. Firstly, LL37 mimetic peptide-W379 peptides were rapidly released to eliminate the bacterial biofilm on the wound. Then, 3D radially aligned nanofiber scaffolds loaded with W379 antimicrobial peptide and PDGF-BB were used to treat the wound to prevent bacterial infection recurrence and promote angiogenesis and granulation tissue regeneration, thereby accelerating wound healing. In the presented study, we found that the combined use of burst and controlled release of W379 antimicrobial peptide effectively clears the bacterial biofilm and prevents the recurrence of bacterial infection. Additionally, we found that the removal of the bacterial biofilm contributed to modulating the local inflammatory response from a pro-inflammatory type to a pro-regenerative type. Furthermore, the use of PDGF-BB significantly promotes neovascularization and granulation tissue regeneration in the wound bed, resulting in accelerating re-epithelialization and wound closure. Our study provides a promising treatment method for the repair of infected chronic wounds.

## Graphical abstract

Graphical Abstract. Preparation and characterization of 3D radially aligned scaffold (RAS) loaded with drug-carrying microspheres. This work reports a two-step strategy for treating infected chronic wounds. Firstly, LL37 mimetic peptide-W379 peptides were rapidly released to eliminate the bacterial biofilm on the wound. Then, 3D radially aligned nanofiber scaffolds loaded with W379 antimicrobial peptide and PDGF-BB were used to treat the wound to prevent bacterial infection recurrence and promote angiogenesis and granulation tissue regeneration, thereby accelerating wound healing.

**Figure:**
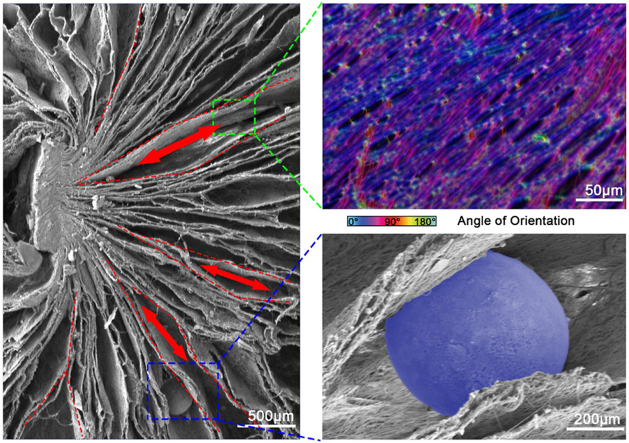

## 1 Introduction

Chronic wounds, including venous leg ulcers (VLU), diabetic foot ulcers (DFU), and pressure ulcers, have considerable prevalence worldwide [1]. Chronic wounds like VLUs affect around 1 % of the population in developed countries, and DFUs affect 15%–25 % of patients with diabetes mellitus [2]. Patients with chronic wounds experience worse mobility and mental health outcomes compared to those without such skin wounds [3]. Additionally, chronic ulcers contribute to a significant economic burden due to their prolonged treatment durations and high recurrence rates. For example, In the UK, the management of chronic wounds costs the National Health Service approximately 5.6 billion pounds annually, accounting for 2–3% of the total healthcare expenditure [4,5]. Similarly, chronic wounds cost about $3.5 billion annually in Australia, representing 2 % of the national healthcare budget [6]. It is evident that chronic wounds not only significantly impact the lives of patients and their families but also impose a substantial economic burden on the government's healthcare system. Therefore, there is an urgent need to discover safe and effective wound repair treatments.

Bacterial infection is a common complication of chronic wounds, which can further delay the healing process. The current clinical treatment strategy involves initial debridement to remove infected tissue, followed by using antibiotics or silver ion dressings to control the infection. However, the use of antibiotics can easily lead to bacterial resistance [7], and silver ions can pose potential cytotoxicity issues, hindering re-epithelialization [8]. In recent decades, significant advancements have been made in developing antibacterial peptides. These peptides are crucial in the fight against multidrug-resistant bacteria. For instance, Wang et al. developed several shorter peptides of human cathelicidin LL-37 derivatives, like KR-12 [9], KR-8 [10], and W379 [11], retaining the antimicrobial properties while minimizing toxicity. In our previous studies, W379 antimicrobial peptides had exhibited good antibacterial effects and biocompatibility [11,12]. However, its drug release model needs to be further improved to prevent the recurrence of bacterial infections.

Another challenge in chronic ulcer healing is the difficulty in forming granulation tissue to fill the defect [3,13]. To address this issue, the platelet-derived growth factor-BB (PDGF-BB) was the first growth factor approved by the U.S. Food and Drug Administration, which primary function is to promote the formation of granulation tissue. For one thing, PDGF-BB stimulates the recruitment and proliferation of endothelial cells, thereby facilitating new vessel formation to ensure an adequate blood supply to the wound site, providing necessary nutrients and oxygen, which are vital for tissue repair and regeneration [14,15]. For another, PDGF-BB promotes fibroblast proliferation and enhances the production of extracellular matrix components such as collagen, which is crucial for wound strength and integrity [16]. After years of verification, PDGF-BB has demonstrated precise efficacy and safety in chronic wound repair.

In the presented study, we used porous 3D radially aligned nanofiber scaffolds (RAS) as substrates, which can quickly recruit many fibroblasts, endothelial cells, and keratinocytes to participate in wound repair [17,18]. Besides, we also aim to encapsulate W379 antibacterial peptides and PDGF-BB into RAS to clear bacterial infections and enhance granulation tissue formation. To achieve this target, we will employ both burst and controlled release modes to achieve rapid onset and long-lasting effects. In addition, we will detect bacterial clearance, granulation tissue formation, and local inflammatory responses.

## 2 Results and discussion

### 2.1 Preparation and characterization of drug-carrying microsphere-loaded RAS

**Fig. 1:**
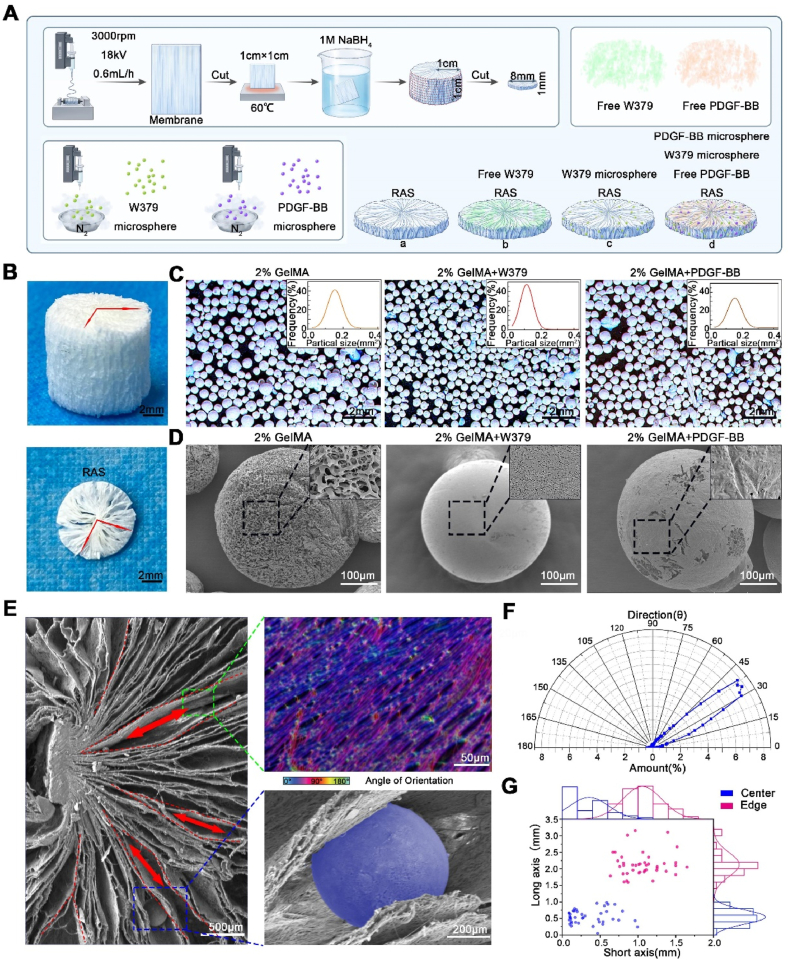
**Preparation and characterization of 3D radially aligned scaffold (RAS) loaded with drug-carrying microspheres**. A) Schematic of the preparation processes of the RAS, and W379 antibacterial peptides/PDGF-BB loaded RAS. B) Photographs of the RAS. C) Stereomicroscope images and particle size distributions of 2 % GelMA microspheres, W379 peptides encapsulated GelMA microspheres, and PDGF-BB encapsulated GelMA microspheres. D) The surface morphologies of 2 % GelMA microspheres, W379 peptides encapsulated GelMA microspheres, and PDGF-BB encapsulated GelMA microspheres. E) SEM images show the hybrid RAS was composed of radially aligned PCL nanofibers with microspheres uniformly distributed inside. F) Characterization of PCL nanofiber arrangement in RAS. G) Quantitative pore size analysis at the RAS center and edge area.

Electrospun nanofibers have been widely used for tissue regeneration [19]. In this study, the 3D radially aligned nanofiber scaffold (RAS) was prepared using modified gas foaming technology [17,20] (Fig. 1A). The macroscopic image of RAS shows its inner porous structure with radial orientation (Fig. 1B). Then, the W379 antibacterial peptides-encapsulated microspheres (W379 microspheres) and PDGF-BB-encapsulated microspheres (PDGF-BB microspheres) were prepared by electrospray, respectively. The average particle size of 0.2 % GelMA microsphere, W379 microspheres, and PDGF-BB microspheres was (0.23 ± 0.10) mm^2^, (0.12 ± 0.04) mm^2^, and (0.18 ± 0.06) mm^2^ (Fig. 1C and D). Finally, free W379 antibacterial peptides, free PDGF-BB, W379 microspheres, and PDGF-BB microspheres were assembled into the RAS for in vitro and in vivo study use (Fig. 1A). As shown in Fig. 1E, the microspheres were evenly dispersed into the internal channels of the RAS. Each layer of nanofiber membrane consists of a mass of aligned PCL nanofibers [18,20]. And the degree of fiber orientation is highly consistent (Fig. 1F). The average length of the long axis and short axis of the nanofiber channel in the central region is 0.544 ± 0.2145 mm and 0.3562 ± 0.2594 mm, and the average length of the long axis and short axis in the surrounding region is 2.13 ± 0.36 mm and 1.057 ± 0.26 mm (Fig. 1G), which is consistent with our previous studies [17]. Meanwhile, we also analyzed the release rates of W379 and PDGF-BB. We found that 73.68 % of W379 could be released within one day through blasting (Figure S1). However, by utilizing microsphere loading, the release cycle of W379 was extended, with 72.34 % being released over 21 days (Figure S2). Similarly, PDGF-bb was rapidly released in the early stage and slowly released in the later stage. The highest release rate occurred on the first day, with 56.49 % of PDGF-BB released by day 21 (Figure S3).

### 2.2 In vitro antibacterial ability of W379 peptides

**Fig. 2:**
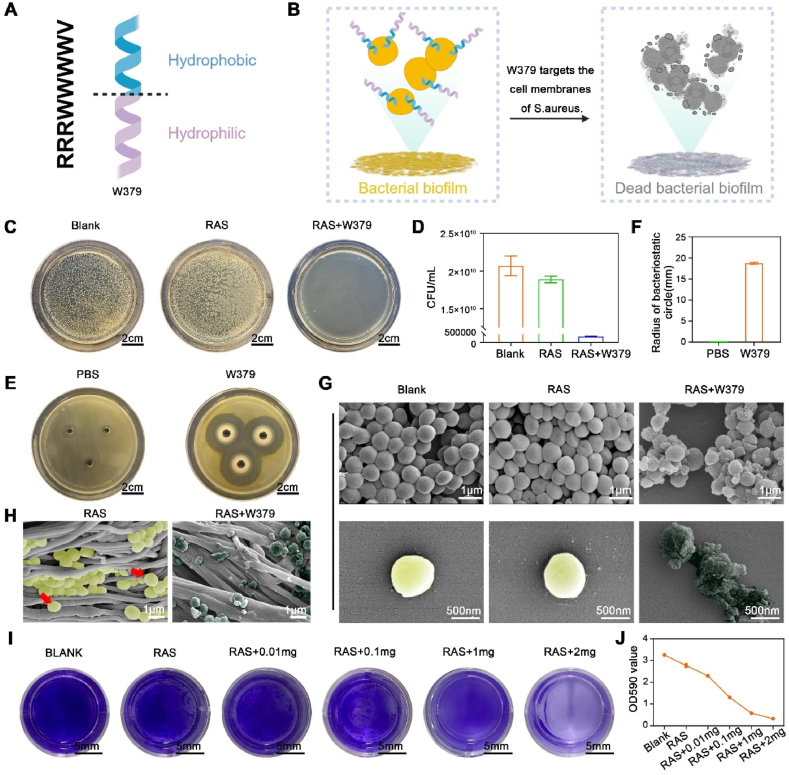
**Antibacterial activities of the W379 peptide in vitro**. A) The scheme shows the amino acid sequence of the W379 antimicrobial peptide is RRRWWWWV, and the short peptide has a hydrophilic amino acids cluster and hydrophobic amino acids cluster in two ends. B) The schematic diagram explains the bactericidal mechanism of W379 antimicrobial peptide, the hydrophobic segment can be inserted into the bacterial membrane, thereby killing bacteria. C, D) Photographs and colony counting of the *S.aureus* colonies after 24 h culture in the RAS and RAS + Free W379 peptide groups. The blank group was used as a control. E, F) Size of the antibacterial zone after incubation with W379 peptide solution for 24 h. G, H) The survival of *S. aureus* in the RAS and RAS + Free W379 peptide groups after 24h of indirect (G) and direct co-culture (H). I, J) Crystal violet staining images of formed *S.aureus* biofilm and its corresponding absorbance in different concentrations of W379 peptide-loaded RAS groups. The blank group was used as a control.

A biofilm is a syntrophic community of bacteria in which cells closely stick to each other. These bacteria are further embedded within a slimy extracellular matrix, resulting in antibacterial drugs that cannot directly act on bacteria. This characteristic makes bacterial biofilms significantly more challenging to treat than regular bacterial infections [21,22]. W379 is a short peptide with eight amino acids. It has a vertical amphiphilic structure with a hydrophobic and hydrophilic end (Fig. 2A) [[23], [24], [25]]. The antibacterial mechanism of the W379 antimicrobial peptide is that its hydrophobic end can be inserted into the bacterial cell membrane [25,26], thereby destroying bacterial biofilms (Fig. 2B). The in vitro bacteriostatic experiment results demonstrated that the RAS + W379 group exhibited a significant bactericidal effect, inhibiting 99.84 % of S. aureus growth. In contrast, the RAS scaffolds alone did not show significant antibacterial effects (Fig. 2C and **D**). In the antibacterial zone experiment, W379 effectively inhibited bacterial growth, with an antibacterial zone radius of 20.25 mm and an inhibition rate of 81.3 % (Fig. 2E and **F**). Typically, an antibacterial ring diameter greater than 7 mm indicates good antibacterial activity. In the indirect contact culture experiments, SEM images showed that the *S. aureus* in the RAS + W379 group was significantly collapsed and deformed compared to the blank and RAS groups (Fig. 2G). In the direct contact culture experiments, SEM images of RAS and RAS + W379 scaffolds co-cultured with *S. aureus* showed that the bacterial membrane on the RAS scaffold was smooth and intact. In contrast, the bacterial membrane on the RAS + W379 scaffold was rough, incomplete, and even broken (Fig. 2H). Subsequently, the biofilm inhibition performance of W379 was evaluated using crystal violet staining. The results showed that as the concentration of W379 increased, biofilm inhibition also increased, with 2 mg of W379 almost completely inhibiting the formation of bacterial biofilm (Fig. 2I and **J**). No new antibiotics have been discovered for a long time in the fight against bacterial infection. Besides W379 peptides, researchers have identified a variety of effective antimicrobial peptides [27,28], which hold promise for addressing bacterial biofilms, drug-resistant bacteria, and even superbugs.

### 2.3 The in vivo healing evaluation of infectious diabetic skin wound

In this work, we used STZ to induce a type 2 diabetes model. After 3 weeks, infectious diabetic skin wounds were made. What we want to emphasize here is that after completing the diabetes modeling, it takes at least 3 weeks for the skin to undergo glycosylation lesions and closely resemble the characteristics of clinical diabetic skin. Such diabetic skin wounds are meaningful. Otherwise, the wounds created will not exhibit the characteristics of chronic wounds.

**Fig. 3:**
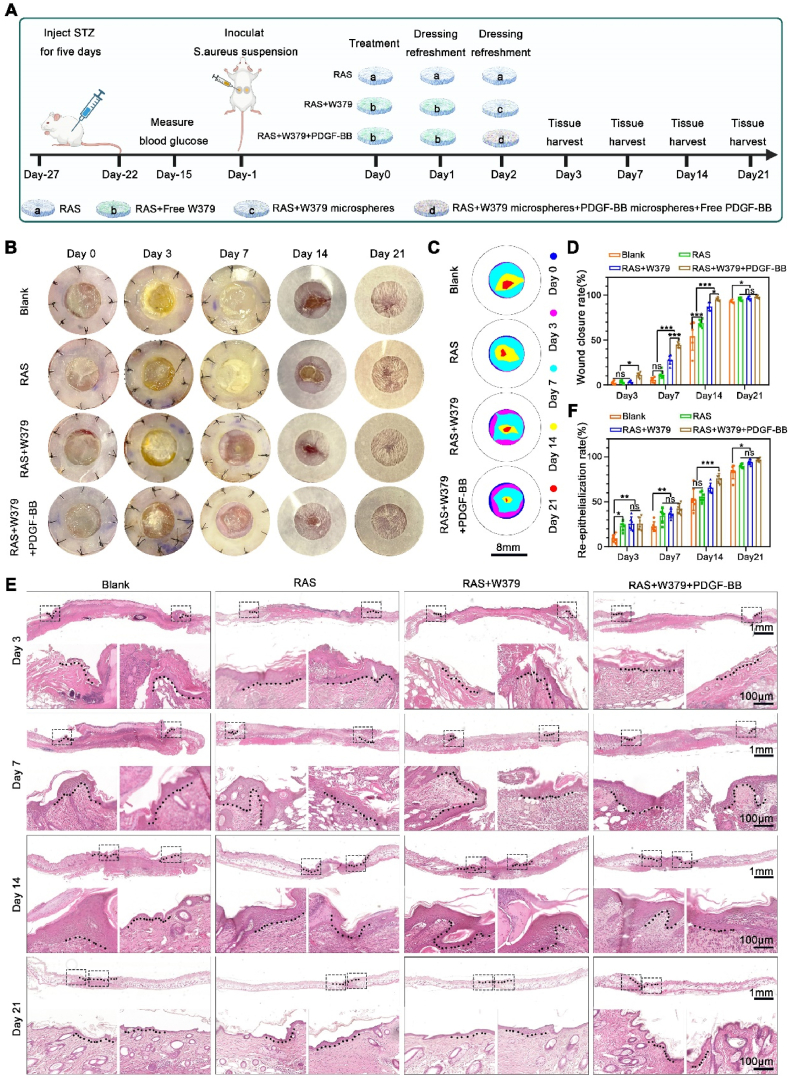
**The in vivo evaluation of wound healing in diabetic mice**. A)The schematic illustrates the establishment of the type-2 diabetes model, the corresponding diabetic skin wound model, and wound management of RAS, RAS + W379, and RAS + W379+PDGF-BB groups, respectively. B) Wound photographs of Blank, RAS, RAS + W379, and RAS + W379+PDGF-BB groups after 0, 3, 7, 14, and 21 days of treatment. C) Traces wound closure at different indicated time points. D) The quantification of wound closure rate at different indicated time points. E) H&E staining of wound area in the Blank, RAS, RAS + W379, and RAS + W379+PDGF-BB groups on day 3, 7, 14, and 21. F) The re-epithelialization rates of the Blank, RAS, RAS + W379, and RAS + W379+PDGF-BB groups on day 3, 7, 14, and 2. *p < 0.05, **p < 0.01, ***p < 0.001.

Each experimental group received different treatments after forming the bacterial biofilm (Fig. 3A). After bacterial biofilm formation, the survival rate of mice in RAS + W379 and RAS + W379+PDGF-BB groups was 100 %, while the survival rate of mice in the control and RAS groups was approximately 85.7 %. This evidence supports that our preparation is a bacterial biofilm infection model, rather than a conventional bacterial infection model [29,30]. As shown in Fig. 3B–**3D**, the wound bed of RAS + W379 and RAS + W379+PDGF-BB groups became clean after 7 days of treatment, and the wounds had begun to heal. After 14 days of treatment, the wounds of RAS + W379+PDGF-BB group had completed closure compared to the other 3 groups. And there was no apparent scar area in the RAS + W379+PDGF-BB group after 21 days of treatment. Following, H&E staining was performed to assess the granulation tissue formation and re-epithelialization. We found the RAS + W379 and RAS + W379+PDGF-BB groups had significant granulation tissue formation after 7 days of treatment, especially in the RAS + W379+PDGF-BB group. Moreover, the RAS + W379+PDGF-BB group showed the most significant re-epithelialization compared to the other groups after 14 days of treatment, with a re-epithelialization rate as high as (81.04 ± 5.47)%. After 21 days of treatment, the RAS + W379+PDGF-BB group nearly completed re-epithelialization, while the other 3 groups still had a gap without epidermal coverage (Fig. 3E and **F**).

### 2.4 In vivo antibacterial ability of W379 peptide

**Fig. 4:**
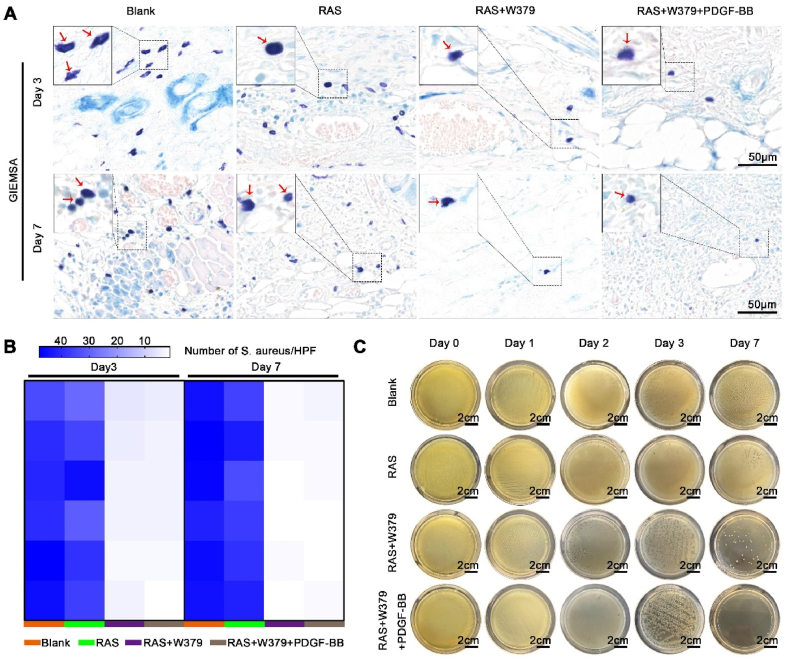
**Bacterial removal in vivo**. A) Giemsa staining in the wound area of the Blank, RAS, RAS + W379, and RAS + W379+PDGF-BB groups after 3 and 7 days of treatment. B) The quantification of detected *S.aureus* in the wound area of the Blank, RAS, RAS + W379, and RAS + W379+PDGF-BB groups after 3 and 7 days of treatment. C) Bacterial culture photographs of skin tissue infected with *S.aureus* biofilm during wound healing.

We also tested the in vivo antibacterial effect of the W379 antimicrobial peptide during the wound healing. Giemsa staining revealed abundant *S. aureus* in the wound tissue of both the Blank and RAS groups on day 3 and day 7. In contrast, the RAS + W379 and RAS + W379+PDGF-BB groups exhibited only trace amounts of residual *S. aureus*. Especially on day 7, there was almost complete absence of bacteria in the wound tissue of the RAS + W379 and RAS + W379+PDGF-BB groups (Fig. 4A and **B**). At the same time, bacteria collected and cultured from the wound surface showed a positive correlation between bacterial numbers and wound healing in the RAS + W379 and RAS + W379+PDGF-BB groups. There were nearly no detectable bacteria on the wounds of these groups after 7 days of treatment (Fig. 4C). In this in vivo study, we combined the use of burst and controlled release of W379 antimicrobial peptide to effectively clear the bacterial biofilm and prevent the recurrence of bacterial infection. Preventing bacterial infections is crucial, especially in clinical practice, where recurrent bacterial infections can lead to the emergence of drug-resistant strains [31]. One significant advantage of antimicrobial peptides over antibiotics is their ability to combat bacteria without promoting the development of drug-resistant bacteria [32].

### 2.5 The proliferative activities detection during wound healing

**Fig. 5:**
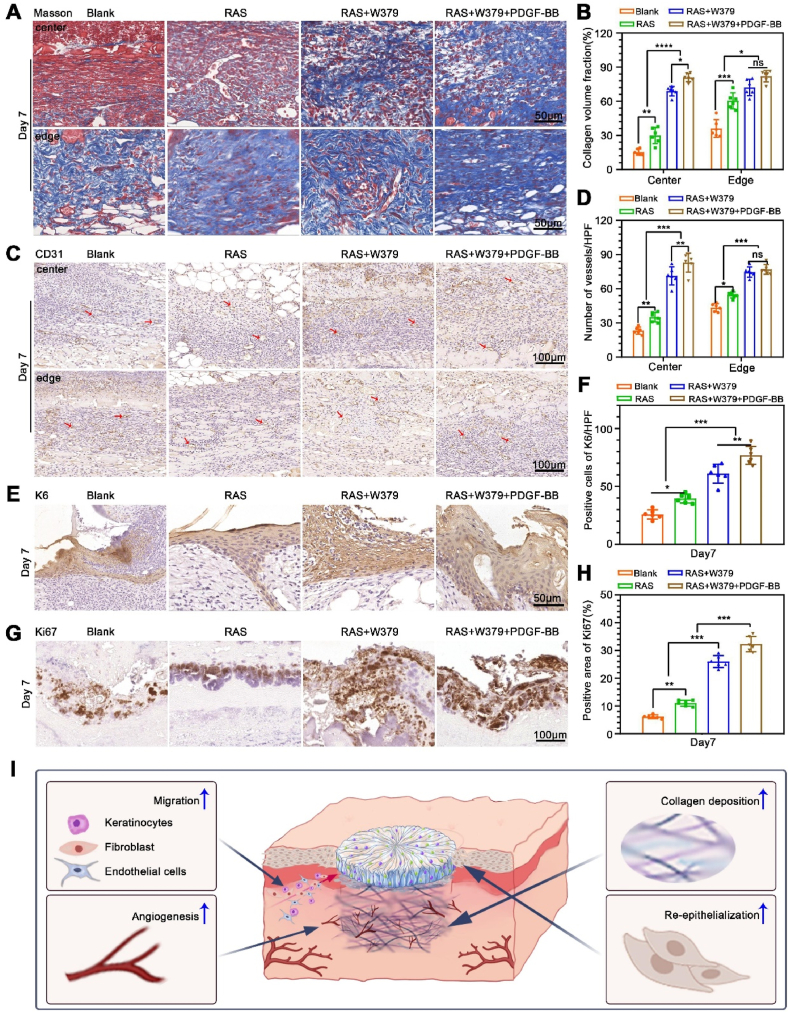
**The proliferative activities detection during wound healing**. A, B) The trichrome staining shows the collagen deposition in the wound central area of the Blank, RAS, RAS + W379, RAS + W379+PDGF-BB groups after 7 days of treatment. C,D) The CD31 immunohistochemical staining of the wound central area in the Blank, RAS, RAS + W379, RAS + W379+PDGF-BB groups after 7 days of treatment. E,F) The K6 immunohistochemical staining of the wound central area in the Blank, RAS, RAS + W379, RAS + W379+PDGF-BB groups after 7 days of treatment. G,H) The Ki67 immunohistochemical staining of the wound central area in the Blank, RAS, RAS + W379, RAS + W379+PDGF-BB groups after 7 days of treatment. I)The schematic illustrates that the treatment of RAS + W379+PDGF-BB is capable of promoting cell recruitment, angiogenesis, collagen deposition, and re-epithelialization based on controlling bacterial infection. *p < 0.05, **p < 0.01, ***p < 0.001, ****p < 0.0001.

After the bacterial biofilm infection is cleared, the wound enters a normal proliferative phase. This study used the free PDGF-BB and PDGF-BB microspheres to promote granulation tissue formation. Masson's trichrome staining revealed significant collagen deposition in the wound area of the RAS + W379 and RAS + W379+PDGF-BB groups compared to control and RAS groups after seven days of treatment (Fig. 5A and **B**). It is mainly because PDGF-BB can promote fibroblast proliferation and enhance collagen deposition [16], and PDGF-BB is the most popular growth factor used in chronic wound repair [14,33]. Immunohistochemical staining of CD31 demonstrated a higher number of new blood vessels in the wound center and surrounding area of the RAS + W379 and RAS + W379+PDGF-BB groups than the blank and RAS groups. Notably, the RAS + W379+PDGF-BB group exhibited the highest number of new blood vessels in the central region (Fig. 5C and **D**). It has been verified that PDGF-BB also can stimulate the recruitment and proliferation of endothelial cells, thereby facilitating new vessel formation [34]. The newly formed blood vessels and deposited collagen fibers together constitute granulation tissues to fill the skin defect. After granulation tissue is formed, keratinocytes begin to proliferate and migrate to complete re-epithelialization [35]. Therefore, we also examined the proliferation of keratinocytes. Immunohistochemical staining for K6 revealed that the expression level of K6 was significantly higher in the RAS + W379+PDGF-BB group compared to the other groups after 7 days of treatment (Fig. 5E and **F**). We also examined the expression of proliferative cell marker Ki67. The results showed that the Ki67-positive area in the RAS + W379 group and RAS + W379+PDGF-BB group was significantly larger than in the other two groups on day 3. On day 7, the RAS + W379+PDGF-BB group exhibited the largest Ki67-positive area (Fig. 5G and **H**). According to the aforementioned results, the schematic illustrates that the treatment of RAS + W379+PDGF-BB is capable of promoting cell recruitment, angiogenesis, collagen deposition, and re-epithelialization based on controlling bacterial infection (Fig. 5I).

### 2.6 Inflammatory response during wound healing

**Fig. 6:**
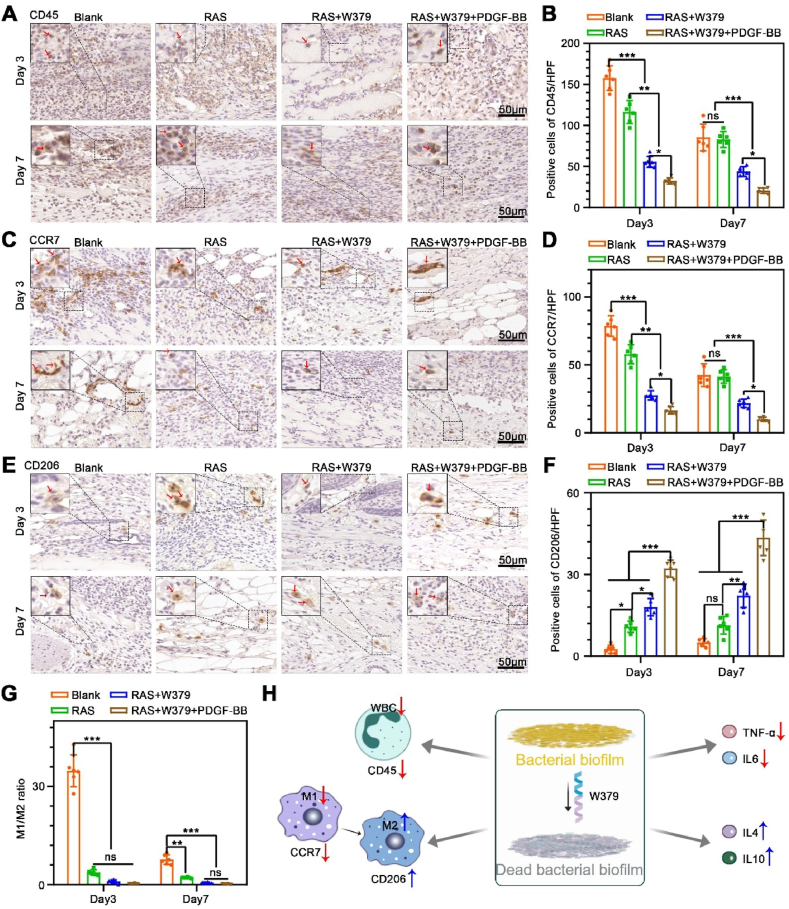
**Inflammatory responses during wound healing**. A,B) The CD45 immunohistochemical staining of the wound central area in the Blank, RAS, RAS + W379 and RAS + W379+PDGF-BB groups after 3 and 7 days of treatment. C,D) The CCR7 immunohistochemical staining of the wound central area in Blank, RAS, RAS + W379 and RAS + W379+PDGF-BB groups after 3 and 7 days of treatment. E,F) The CD206 immunohistochemical staining of the wound central area in the Blank, RAS, RAS + W379 and RAS + W379+PDGF-BB groups after 3 and 7 days of treatment. G) The M1/M2 ratio in the Blank, RAS, RAS + W379 and RAS + W379+PDGF-BB groups. H) Response of W379 antimicrobial peptide to wound inflammatory cells and inflammatory cytokine after bacterial biofilm clearance. *p < 0.05, **p < 0.01, ***p < 0.001.

**Fig. 7:**
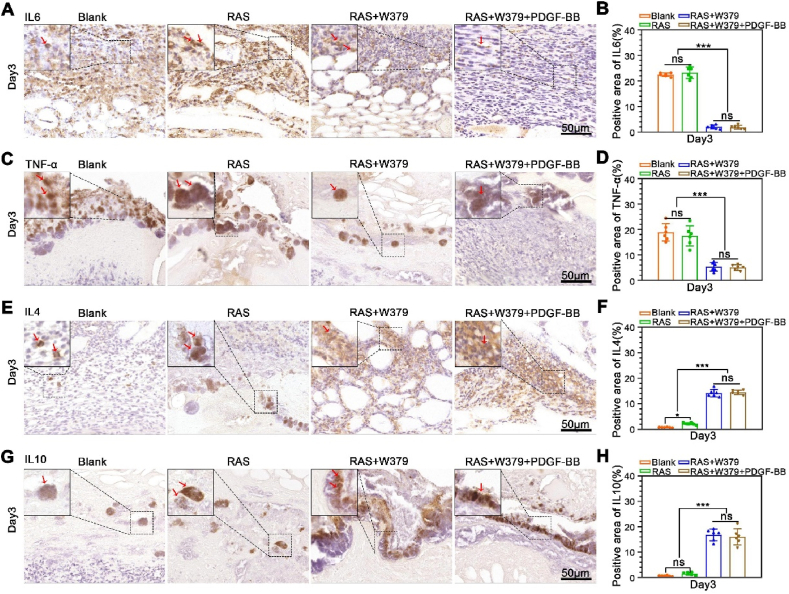
**The expression of inflammatory and anti-inflammatory factors during wound healing**. A, B) The Immunohistochemical staining and quantification of IL-6 expression in the wound central area of Blank, RAS, RAS + W379, RAS + W379+PDGF-BB groups after 3 days of treatment. C, D) The immunohistochemical staining and quantification of TNF-α expression in the wound central area of the Blank, RAS, RAS + W379, RAS + W379+PDGF-BB groups after 3 days of treatment. E, F) The immunohistochemical staining and quantification of IL-4 expression in the wound central area of the Blank, RAS, RAS + W379, RAS + W379+PDGF-BB groups after 3 days of treatment. G, H) The immunohistochemical staining and quantification of IL-10 expression in the wound central area of the Blank, RAS, RAS + W379, RAS + W379+PDGF-BB groups after 3 days of treatment. *p < 0.05, **p < 0.01, ***p < 0.001.

When the bacterial biofilm on the wound surface is cleared, the local inflammatory response in the wound bed also changes [36]. Therefore, we also detected the expression of inflammatory cells and inflammatory factors in the wound bed. The CD45 immunohistochemical staining of RAS + W379 and RAS + W379+PDGF-BB groups was significantly reduced compared with the control and RAS groups (Fig. 6A and **B**). In addition, compared to the control group, the expression of the M1 macrophage marker CCR7 decreased in the RAS + W379 and RAS + W379+PDGF-BB groups on day 3 and day 7 (Fig. 6C and **D**), while the expression of the M2 macrophage marker CD206 increased (Fig. 6E and **F**). Concurrently, we observed that the M1/M2 ratio in the RAS + W379 group and RAS + W379+PDGF-BB group was lower than 1 on both day 3 and day 7, indicating the local inflammatory response favoring a pro-regenerative status [37] (Fig. 6G). Moreover, we also examined the expression of inflammatory and anti-inflammatory factors in the early stage of wound healing. We found the expression of the IL-6 and TNF-α in the RAS + W379 and RAS + W379+PDGF-BB groups were significantly lower than those in the blank and RAS groups (Fig. 7A–**7D**). Conversely, the expression of IL-4 and IL-10 in the RAS + W379 and RAS + W379+PDGF-BB groups was significantly increased compared to the blank and RAS groups (Fig. 7E–**7H**). According to the aforementioned results, we speculate that after W379 antimicrobial peptides clear bacterial biofilms, they help transform the local inflammatory response in the wound, decreasing M1 macrophage polarization and pro-inflammatory cytokine expression while increasing M2 macrophage polarization and anti-inflammatory cytokine expression (Fig. 6H).

### 2.7 Histocompatibility evaluation

**Fig. 8:**
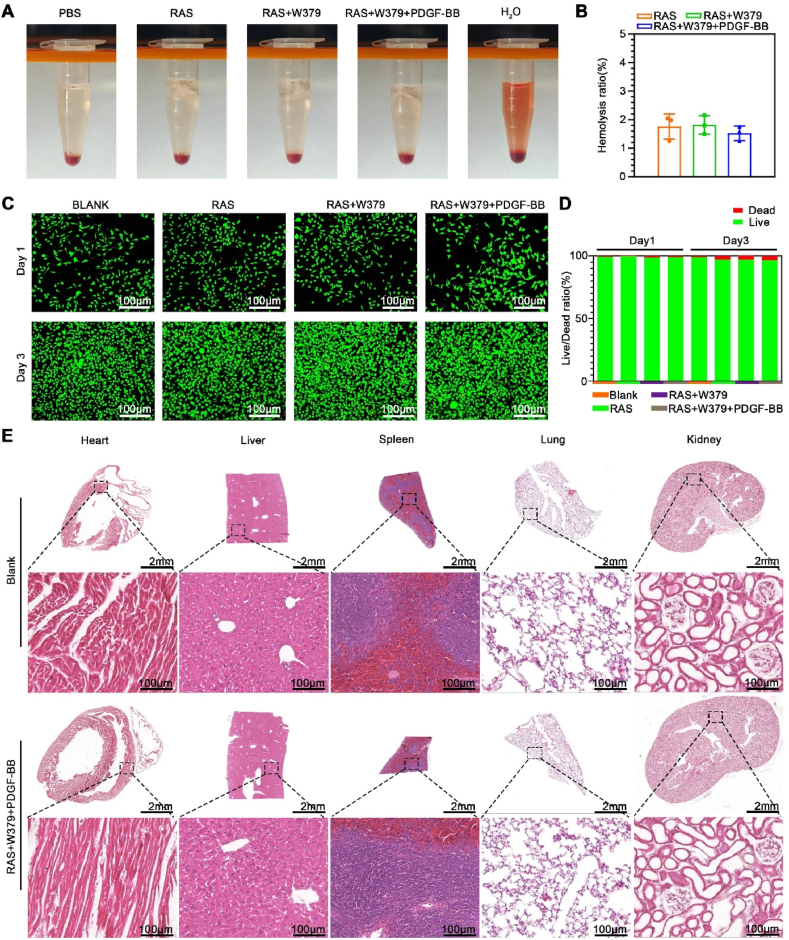
**Histocompatibility test of W379 antibacterial peptide**. A, B) Hemolysis test of the RAS, RAS + W379, RAS + W379+PDGF-BB scaffolds. C, D) The viability of L929 cells treated with extraction solution of RAS, RAS + W379, RAS + W379+PDGF-BB scaffolds for 1 and 3 days. E) Histological observations of different organs (heart, liver, spleen, lung, and kidney) after 21 days of treatment in the Blank, RAS + W379+PDGF-BB group.

In our experiments, W379 peptides have demonstrated excellent capabilities in removing bacterial biofilms. However, due to its nature as an amphipathic short peptide, it may have potential cytotoxicity [38]. Therefore, we further evaluated the blood compatibility, cell compatibility, and tissue compatibility of the W379 antimicrobial peptide. The blood compatibility of the W379 loaded scaffold was assessed by measuring the hemolysis of mouse red blood cells. After a 1 h incubation, almost no hemoglobin was released in the experimental group, with hemolysis rates all below 5 %, indicating good blood compatibility (Fig. 8A and **B**). Following, the viability of L929 cells treated with extraction solution of RAS, RAS + W379, RAS + W379+PDGF-BB scaffolds for 1 and 3 days and then stained with Calcein-AM and PI. The results showed that the RAS group, RAS + W379 group, and RAS + W379+PDGF-BB group all exhibited the same growth trend as the Blank group. After 1 day, the cell survival rate of all groups was over 98 %. After 3 days, the cell survival rate of all groups remained above 96 % (Fig. 8C and **D**). Finally, after 21 days of treatment, the blank group and the RAS + W379+PDGF-BB group had no significant changes in the heart, liver, spleen, lung, and kidney. This means that the W379 peptides did not significantly damage the vital organs of the mice after long-term wound treatment (Fig. 8E).

## 3 Conclusion

In the presented study, we developed a W379 antibacterial peptide and PDGF-BB co-loaded 3D radially aligned nanofiber scaffold for treating infected chronic wounds. Firstly, LL37 mimetic peptide-W379 peptides were rapidly released to eliminate the bacterial biofilm on the wound. Then, 3D radially aligned nanofiber scaffolds loaded with W379 antimicrobial peptide and PDGF-BB were used to prevent bacterial infection recurrence and promote angiogenesis and granulation tissue regeneration, thereby accelerating wound healing. In the presented study, we found that the combined use of burst and controlled release of W379 antimicrobial peptide effectively clears the bacterial biofilm and prevents the recurrence of bacterial infection. Additionally, we found that the removal of the bacterial biofilm contributed to modulating the local inflammatory response from a pro-inflammatory type to a pro-regenerative type. The expression of M1-type macrophages and pro-inflammatory cytokines (IL-6 and TNF-α) was decreased, while the expression of M2-type macrophages and anti-inflammatory cytokines (IL-4 and IL-10) was increased. Furthermore, the use of PDGF-BB significantly promotes neovascularization, collagen deposition, and granulation tissue regeneration in the wound bed, resulting in accelerating re-epithelialization and wound closure. The presented study provides a promising treatment method for the repair of infected chronic wounds.

However, this study does have some limitations. For instance, clinical infectious chronic wounds are much more complex than the animal models we developed. Most patients experience multiple bacterial biofilm infections. In future experiments, we plan to establish a wound model that includes multiple bacterial biofilm infections.

## 4 Experimental section

### 4.1 Materials and reagents

Polycaprolactone (MW: 80 kDa) and pluronic F-127 were ordered from Sigma-Aldrich (St. Louis, USA). Gelatin methacryloyl (GelMA) was purchased from Wenzhou Shuhe Biological Technology Co.,ltd (Zhejiang, China). Sodium borohydride (catalog no. S432209) was purchased from Aladdin (Shanghai, China). Tryptic soy broth (TSB) bacterial medium was purchased from Thermo Fisher Scientific Oxoid. Penicillin-streptomycin (catalog no. 15140122), fetal bovine serum (catalog no.10099-141C), and Dulbecco's modified eagle medium (catalog no. 11965118) were obtained from Gibco (Shanghai, China). FITC-BSA (catalog no. bs-0292P-FITC) was purchased from Bioss (Beijing, China). Modified giemsa staining solution (catalog no. C0131), crystal violet staining solution (catalog no. C0121), and Calcein AM/PI Cell Viability/Cytotoxicity Assay Kit (catalog no. C2015) was purchased from Beyotime (Shanghai, China). Anti-CD31 rabbit pAb (catalog no. ab28364), anti-CD45 rabbit pAb (catalog no. ab10558), anti-Ki67 rabbit pAb (catalog no. ab16667), and anti- CCR7 rabbit pAb (catalog no. ab253187) were ordered from Abcam (Cambridge, UK). Anti-K6 rabbit pAb (catalog no. bsm-60235R) was ordered from Bioss (Beijing, China). Anti-CD206 rabbit pAb (catalog no. PA5-101657) was ordered from Invitrogen (California, USA). Anti-IL4 rabbit pAb (catalog no. TA5142M), anti-IL6 rabbit pAb (catalog no. TD6084), anti-IL10 rabbit pAb (catalog no. TD6894), and anti- TNF-α rabbit pAb (catalog no. TA7014) was ordered from Abmart (Shanghai, China). Peptides (95 % pure) were purchased from Shanghai Science Peptide Biological Technology Co.,ltd (Shanghai, China). The Human PDGF-BB ELISA Kit(catalog no. EK0956) was ordered from BOSTER (Wuhan, China).

### 4.2 Preparation of RAS: preparation of RAS

Firstly, a 1 mm thick oriented nanofiber pad is formed by electrospinning with 10 % polycaprolactone (PCL) and 1 % Pluronic F-127 solution. After the nanofiber pad is formed, it is cut into small squares of 1 by 1 cm in liquid nitrogen at a low temperature. Then the side perpendicular to the direction of the nanofiber is heated and fixed, and only one side is fixed. Soak a small square with one side heated and fixed in 1M sodium borohydride solution. The small square will expand in the solution centered on the side that is heated and fixed, and eventually rotate and expand into a cylinder. The cylinder are washed with water. To improve the mechanical properties of the scaffold, the scaffold is soaked in 1 % gelatin solution and freeze-dried. Finally, the freeze-dried cylinders were cut into 1 mm thick sheet scaffolds, which were then fabricated into RAS (radially aligned scaffold) with a diameter of 8 mm using punch biopsy.

### 4.3 Preparation of microspheres

Dissolve 1 mg W379 in 10 mL of solution containing 0.2 % GelMA so that the concentration of W379 is 0.1 μg μL−^1^. Dissolve 50 μg PDGF-BB in 0.5 mL of solution containing 0.2 % GelMA so that the concentration of PDGF-BB is 0.1 μg μL−^1^. The control group was set to 10 mL containing 0.2 % GelMA solution. The three solutions were prepared into microspheres by electrostatic spray technology with a voltage of 12 kV, a flow rate of 2 ml h^−1^, and a distance of 20 cm between the needle and the receiver. The received microspheres are quickly freeze-dried for 24 h.

### 4.4 Preparation of different kinds of RAS

RAS + free W379: Dissolve W379 in a solution containing 0.2 % Gelma so that the concentration of free W379 is 40 μg μL−^1^, and then apply 50 μL free W379 solution to RAS. RAS + W379 microspheres: W379 microspheres containing 10 μg W379 are placed on RAS and 50 μL 0.2 % GelMA solution is added to achieve the effect of fixing the microspheres. RAS + W379 microspheres + PDGF-BB microspheres + free PDGF-BB: W379 microspheres containing 10 μg W379 and PDGF-BB microspheres containing 0.5 μg PDGF-BB were placed on RAS, and finally PDGF-BB was dissolved in 0.2 % GelMA solution, so that the concentration of free PDGF-BB was 0.01 μg μL−^1^. Then 50 μL free PDGF-BB solution was applied to RAS. The above three scaffolds are freeze-dried for 24 h after completion.

### 4.5 SEM analysis of RAS and microspheres

The scaffold, 0.2 % GelMA microspheres, W379 microspheres, and PDGF-BB microspheres were sputtered by Pt for 1 min (High Vacuum Ion Sputtering Instrument, Leica, EM ACE600), and then imaging by SEM (Field Emission Scanning Electron Microscopy, Hitachi, SU8010) at 5 kV acceleration voltage. Finally, through the analysis of the obtained SEM images, the nanofiber orientation and average pore diameter of the scaffold were quantified.

### 4.6 Stereomicroscope morphological analysis of microspheres

The samples of 0.2 % GelMA microspheres, W379 microspheres and PDGF-BB microspheres were photographed under the Stereomicroscope, and the particle sizes of the three microspheres were quantified.

### 4.7 In vitro antibacterial test

RAS and RAS + free W379 were incubated with 10 mL of *S.aureus* (10^5^ CFU mL^−1^) respectively on an shaker for 24 h. The bacterial suspension is dispersed on the surface of the agar plate in gradient to form bacterial colony units. Then count the bacterial colonies.

### 4.8 Inhibition zone test

*S.aureus* suspension (10^5^ CFU mL^−1^) was inoculated on the agar plate, a hole with a diameter of 8 mm was made on the agar plate, and 50uL 40 mg mL^−1^ W379 solution were added to the hole, PBS served as the control group. Incubate at 37 °C for 24 h. After culture, the radius of the growth inhibition zone was measured.

### 4.9 Bacterial SEM detection

In the above in vitro antibacterial effect test, the bacterial suspension and the scaffolds were cleaned with PBS (pH 7.0) for three times, 15 min each time, and centrifuged at 5000 rpm for 3 min to remove the supernatant. Then add 1 ml of 2.5 % glutaraldehyde solution, shake gently and mix thoroughly, suspend the bacterial solution, and fix at 4 °C for 4 h. After 4 h, the bacterial suspension and the scaffolds were centrifuged at 5000 rpm for 3 min, then the supernatant was removed, and the samples were rinsed with PBS for three times for 15 min each time. Dehydrate with ethanol solution with gradient concentration (including 20 %, 30 %, 50 %, 80 %, 90 %, 95 %, 100 % seven concentrations), treat each concentration for 15 min, and centrifuge at 5000 rpm for 3min. Finally, 200uL pure acetone was added, 10 μL suspension was added to clean titanium (5 × 5 × 0.1 mm^3^), and bacteria and scaffolds were observed by SEM.

### 4.10 Inhibition of bacterial biofilm formation

Primarily, 500 μL *S.aureus* (10^6^ CFU^−1^) suspension with pH 5.5 was added to the 24-well plate. Then 500 μL of TBS solution soaked by RAS and RAS + free W379 scaffold was added, wherein the content of W379 in RAS + free W379 scaffold was 0.01 mg, 0.1 mg, 1 mg, 2 mg. After incubation at 37 °C for 2 days, the plate was gently washed with PBS 3 times, and 300 μL 1 % (v/v) crystal violet ethanol solution was added for 15 min. Then a camera was used to image the crystal violet stained plates and measure the absorbance of the crystal violet solution at 590 nm to investigate the ability of W379 to inhibit biofilm formation. In the absence of a scaffold, *S.aureus* suspension with a pH of 5.5 was used as a control.

### 4.11 In vivo bacterial biofilm efficacy test

*S.aureus* was cultured overnight in TSB. Subsequently, 100 μL *S.aureus* strain was transferred into 4 mL fresh TSB medium, cultured for 3 h, and washed with PBS for 3 times. Then adjust the concentration of bacteria to 1 × 10^8^ CFU mL^−1^ and store in 4 °C before use. Female ICR mice aged 8–10 weeks were anesthetized with a mixture of 2 % isoflurane and 1 % oxygen and placed on a heating pad to maintain body temperature. A 4 × 4 cm^2^ area was shaved off the back of the mice, then the area was disinfected three times with iodophor solution and wiped three times with an alcohol cotton. Next, on the back of each mouse, a full-layer excision wound with a diameter of 8 mm was created on each side of the spine with a hole punch (each wound was 0.5–1 cm away from the spine). The wound was fixed with silicone fixation ring to made an anti-contraction model. Immediately after surgery, 10 μL *S.aureus* suspension was inoculated. After 1 day, the infected skin wound model was successfully established, and the mice were randomly divided into 4 groups: Blank group, RAS group, RAS + W379 group, RAS + W379+PDGF-BB group (n = 3 in each group). Among them, the Blank group did not do any treatment, and the RAS group wound covered the RAS and replace new RAS every 24h in first 3 days. The RAS + W379 group and the RAS + W379+PDGF-BB group first used the RAS + free W379 scaffold, and replaced it with a new RAS + free W379 scaffold every 24 h in first two days. On the third day, RAS + W379 group was replaced with RAS + W379 microsphere scaffold, and RAS + W379+PDGF-BB group was replaced with RAS + W379 microsphere + PDGF-BB microsphere + free PDGF-BB scaffold. After each scaffold implantation, the wound is covered with a sterile dressing. The wounds were observed daily and photographed on days 3, 7, 14 and 21. Tissue samples were taken at these time points. Calculation of wound closure rate: Wound closure rate (%) = (Si-St)/Si × 100 %. Si represents the initial wound area, and St represents the wound area at the 3, 7, 14, and 21 days.

### 4.12 Histological observation

The wound tissue was collected at 3, 7, 14 and 21 days after injury and analyzed. The collected wound tissue was fixed with 4 % paraformaldehyde solution, then dehydrated with alcohol gradient, clarified with xylene, and finally embedded with paraffin. Subsequently, the wax block was cut into slices with a thickness of 5 μm. HE staining and Masson trichromatic staining were performed for each group of slices. The re-epithelialization rate was calculated by analyzing the results of HE staining: (distance of re-epithelialization/total width of initial wound) × 100 %

### 4.13 Immunohistochemical staining

The sections of each group were subjected to routine dewaxing, rehydration, antigen extraction and 5 % goat bovine serum albumin solution for 1h. Then, each group of slices were incubated with primary antibodies at 4 °C overnight, including CD45 (1:300), CCR7 (1:300), CD206 (1:300), Ki67 (1:300), CD31 (1:200), K6 (1:300), IL4 (1:250), IL6 (1:250), IL10 (1: 250) and TNF-α (1:250). The sections were treated with HRP-conjugated secondary antibody at room temperature for 1 h. The staining reaction was performed by DAB (diaminobenzidine, 1:20). Finally, the sections were scanned and analyzed using a pathological biopsy scanner.

### 4.14 In vivo antibacterial test

After the establishment of an infectious bacterial biofilm wound. Wound exudates were collected with swabs at days 0, 1, 2, 3, and 7 and were cultured on agar plates for 24 h before plate photographs were taken of each group. Giemsa staining was performed on the wound tissues to evaluate the residual bacteria in the wound.

### 4.15 In vitro cytotoxicity test

The cytotoxicity of each group of scaffolds was evaluated by leaching method. The UV-sterilized scaffolds were soaked in the medium for 24 h. L929 cells (4 × 10^3^ cells/well) were inoculated in 96-well plates for 24 h. 100 μL scaffold leaching solution was used to replace the culture medium. After 1 day and 3 days in the leaching solution, L929 cells were incubated with Calcein/AM and PI at 37 °C for 30 min. Live (green) and dead (red) cells were observed with inverted fluorescence microscopy to evaluate the toxicity of the scaffold to L929 cells.

### 4.16 Hemolysis test

Red blood cells (RBC) were isolated from whole blood of mice mixed with sodium citrate (9:1 ratio) for hemolysis test. The red blood cells were centrifuged at 3000 rpm for 10 min, the supernatant was removed, and the red blood cells were washed three times with PBS. Preparing the 5 % (v/v) RBC suspension. The scaffolds sterilized by ultraviolet were washed 3 times with PBS, and 0.5 mL PBS with scaffolds was incubated with 0.5 mL RBC suspension at 37 °C for 1h, centrifuged at 1000 rpm for 10 min, and 200 μL supernatant was transferred to 96-well plates. Absorbance was measured at 540 nm. Deionized water and PBS were positive and negative controls, respectively. The formula for calculating the hemolysis rate is: Hemolysis rate (%) = (ODs-ODp)/(ODh-ODp) × 100 %, where ODs is the absorbance of the sample, ODh is the absorbance of the deionized water, and ODp is the absorbance of PBS.

### 4.17 Systemic toxicity of scaffolds

In order to evaluate the possible systemic toxicity of RAS + W379+PDGF-BB group, major organs of mice were collected on wounds and treated with scaffolds for 21 days, and healthy mice was used as control. The harvested heart, liver, spleen, kidney and lung tissues were fixed with 4 % paraformaldehyde solution. Finaly, paraffin sections were performed and HE staining was used to evaluate the effects of scaffold on heart, liver, spleen, lung and kidney.

### 4.18 In vitro release experiment of W379 and PDGF-BB

Firstly, RAS + free W379, RAS + W379 microspheres and RAS + W379 microspheres + PDGF-BB microspheres + free PDGF-BB scaffold were immersed in 10 mL PBS (pH = 7) respectively. Remove a certain volume of supernatant at different times. At the same time, add the same volume of supernatant. Finally, the release concentration of W379 was detected by NanoDrop (DeNovix, DS-11), and the release concentration of PDGF-BB was detected by Human PDGF-BB ELISA Kit.

### 4.19 Statistical analysis

All statistical results are expressed as the mean plus standard deviation (SD). The data were analyzed by one-way ANOVA with multiple comparisons. Finally, GraphPad Prism 8.0 software was used to create graphs. A p-value of ≤0.05 was considered as significant.

## CRediT authorship contribution statement

**Fei Li:** Methodology, Investigation, Formal analysis. **Chuwei Zhang:** Investigation, Formal analysis, Data curation. **Xiaoping Zhong:** Investigation. **Bo Li:** Methodology, Investigation. **Mengnan Zhang:** Investigation. **Wanqian Li:** Investigation, Formal analysis. **Lifei Zheng:** Project administration, Resources. **Xinghua Zhu:** Resources, Project administration, Investigation, Formal analysis. **Shixuan Chen:** Writing – review & editing, Writing – original draft, Supervision, Project administration. **Yi Zhang:** Writing – original draft, Resources, Project administration.

## Declaration of Competing interest

All authors declare no conflicts of interest.

## Data Availability

Data will be made available on request.
