# Supplementary material for: A 3D radially aligned nanofiber scaffold co-loaded with LL37 mimetic peptide and PDGF-BB for the management of infected chronic wounds
Source: Mater Today Bio. 2024 Sep 12;28:101237. doi: 10.1016/j.mtbio.2024.101237 (PMC11419797; doi:10.1016/j.mtbio.2024.101237)
Supplement: Multimedia component 1 [file mmc1.docx]

**Supporting Information**

**A 3D radially aligned nanofiber scaffold co-loaded with LL37 mimetic peptide**

**and PDGF-BB for the management of infected chronic wounds**

Fei Li ^1,2 †^, Chuwei Zhang ^1,2 †^, Xiaoping Zhong ^3^, Bo Li ^1,2^, Mengnan Zhang ^1,2^, Wanqian Li ^1,2^, Lifei Zheng ^2^, Xinghua Zhu ^1^*, Shixuan Chen ^2^*, Yi Zhang ^1^*

^1^ Department of Burn and Plastic Surgery, Affiliated Hospital of Nantong University, Nantong 226001, China

^2^ Zhejiang Engineering Research Center for Tissue Repair Materials, Wenzhou Institute, University of the Chinese Academy of Sciences, Wenzhou, Zhejiang 325000, China

^3^ Department of Nursing, Third Affiliated Hospital of Guangzhou Medical University, Guangzhou, Guangdong 510000, China.

^†^ F. Li and C. Zhang contributed equally to this work.

*Corresponding Author:

[zplasty@163.com](mailto:zplasty@163.com) (X. Zhu);

[chensx@wiucas.ac.cn](mailto:chensx@wiucas.ac.cn) (S. Chen);

[zhangyi@ntu.edu.cn](mailto:zhangyi@ntu.edu.cn) (Y. Zhang);


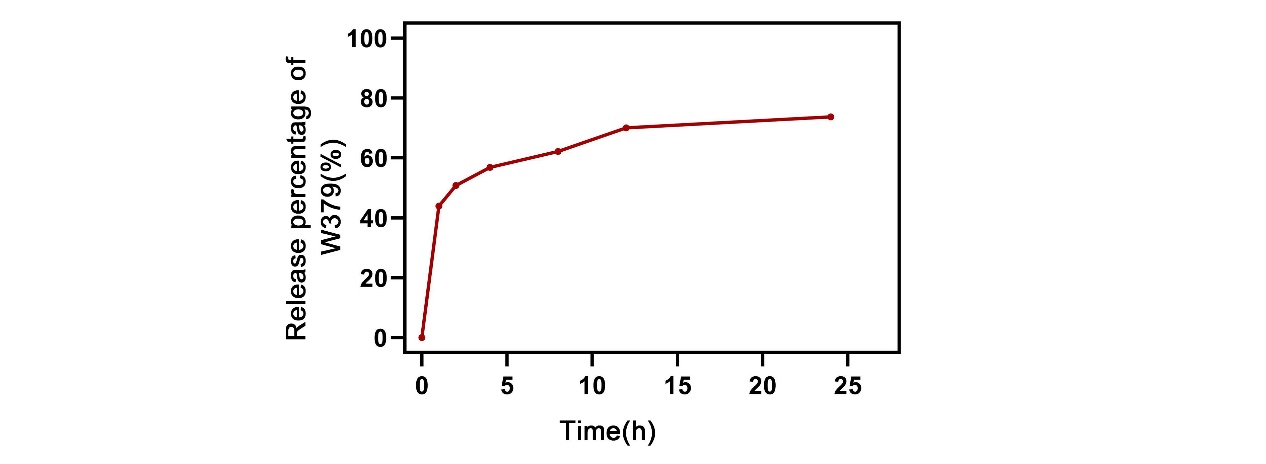


**Figure S1.** The release curve of W379 from RAS + free W379.


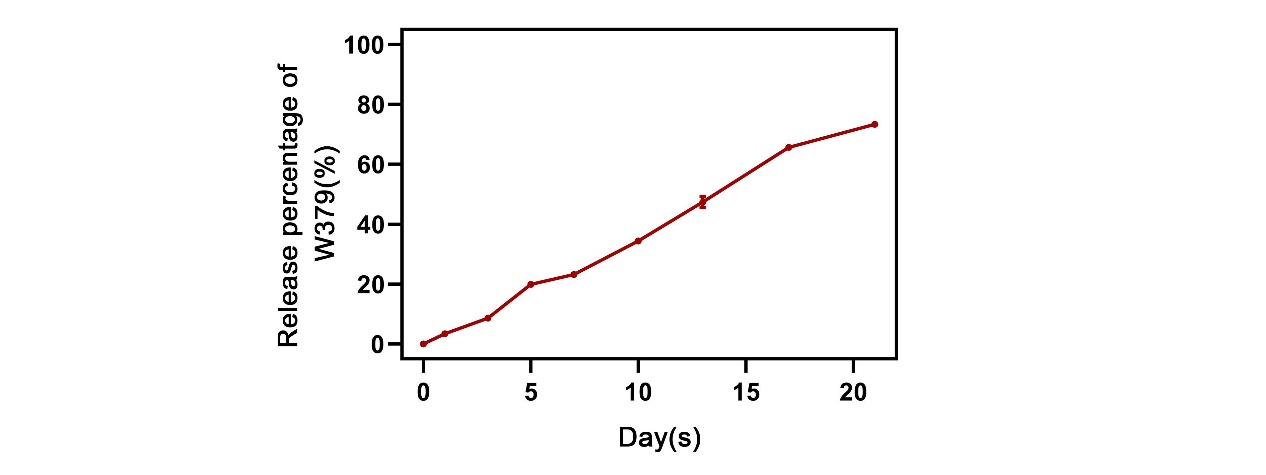


**Figure S2.** The release curve of W379 from RAS + W379 microspheres.


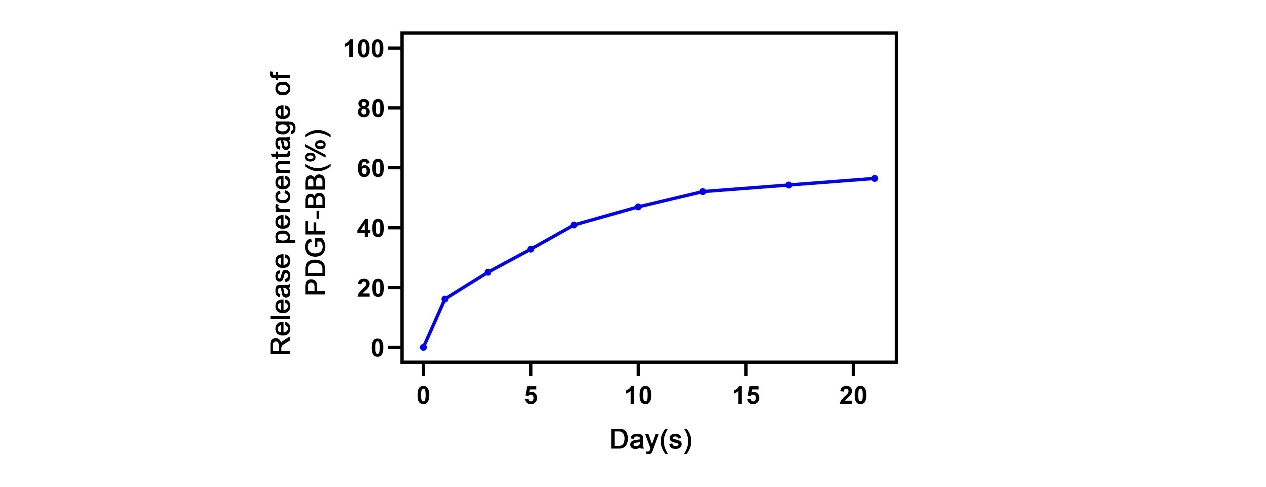


**Figure S3.** The release curve of PDGF-BB from RAS + PDGF-BB microspheres + free PDGF-BB.
